# New framework for automated article selection applied to a literature review of Enhanced Biological Phosphorus Removal

**DOI:** 10.1371/journal.pone.0216126

**Published:** 2019-05-09

**Authors:** Minh Nguyen Quang, Tim Rogers, Jan Hofman, Ana B. Lanham

**Affiliations:** 1 Water Innovation and Research Centre, Department of Chemical Engineering, University of Bath, Bath, United Kingdom; 2 Centre for Networks and Collective Behaviour, Department of Mathematical Sciences, University of Bath, Bath, United Kingdom; Aix-Marseille Universite, FRANCE

## Abstract

**Aims:**

Enhanced Biological Phosphorus Removal (EBPR) is a technology widely used in wastewater treatment to remove phosphorus (P) and prevent eutrophication. Establishing its operating efficiency and stability is an active research field that has generated almost 3000 publications in the last 40 years. Due to its size, including over 119 review articles, it is an example of a field where it becomes increasingly difficult to manually recognize its key research contributions, especially for non-experts or newcomers. Therefore, this work included two distinct but complementary objectives. First, to assemble for the first time a collection of bibliometric techniques into a framework for automating the article selection process when preparing a literature review (section 2). Second, to demonstrate it by applying it to the field of EBPR, producing a bibliometric analysis and a review of the key findings of EBPR research over time (section 3).

**Findings:**

The joint analysis of citation networks, keywords, citation profiles, as well as of specific benchmarks for the identification of highly-cited publications revealed 12 research topics. Their content and evolution could be manually reviewed using a selection of articles consisting of approximately only 5% of the original set of publications. The largest topics addressed the identification of relevant microorganisms, the characterization of their metabolism, including denitrification and the competition between them (Clusters A-D). Emerging and influential topics, as determined by different citation indicators and temporal analysis, were related to volatile fatty acid production, P-recovery from waste activated sludge and aerobic granules for better process efficiency and stability (Clusters F-H).

**Conclusions:**

The framework enabled key contributions in each of the constituent topics to be highlighted in a way that may have otherwise been biased by conventional citation-based ranking. Further, it reduced the need for manual input and *a priori* expertise compared to a traditional literature review. Hence, in an era of accelerated production of information and publications, this work contributed to the way that we are able to use computer-aided approaches to curate information and manage knowledge.

## 1 Introduction

Eutrophication—the over-abundance of certain nutrients in water bodies, unbalancing local ecosystems—is a major environmental concern. To prevent this, numerous technologies have been developed to remove phosphorus from wastewater [1]. Of these technologies, Enhanced Biological Phosphorus Removal (EBPR) is perhaps one of the most popular choices in wastewater treatment plants (WWTP), especially for those with larger capacities. It is essentially a variation of conventional Activated Sludge (AS). By engineering alternating anaerobic, aerobic and often anoxic conditions, the resulting community of microorganisms removes phosphorus (P) by intra-cellular accumulation. Primarily, EBPR offers an alternative to chemical precipitation for P-removal. However, it also became an extremely interesting model of microbial ecology applied to complex engineered environmental systems.

Since its inception in 1975 [2], close to 3000 articles have been published on EBPR and the field continues to grow with many emerging questions (*e.g*., the most recent paper by Barnard and colleagues in 2017 [3]). Almost 40 years later and with such a rich and multidisciplinary body of work [4], it is an example of a field where it becomes increasingly time-consuming for a non-expert or a newcomer (*e.g*., a new PhD student or postdoctoral researcher) to manually review all the existing literature and identify key research highlights, as well as new opportunities for further research. With advances in bibliometric techniques, *i.e*., the statistical analysis of literature, in particular via citation analysis, it is now timely and possible to develop for the first time a suitable bibliometric framework that can systematically identify EBPR’s key areas of research and/or publications. The aim of this framework is to reduce the manual intervention needed to review a specific research area, in this case applied to EBPR.

Such a framework can be interesting to EBPR scientists but also to anyone exploring other research fields and searching for ways to reduce manual input when conducting a literature review. In addition, the resulting bibliometric analysis and review of EBPR’s key areas of research, exemplifies what sort of information can be obtained. While primarily intended for researchers new to the field, the framework may also prove useful to specialists already knowledgeable of the available literature. Structuring the information in a manner that is unbiased (or at least less so) by the authors themselves or sources they typically consult provides a perspective on the whole research area. They may also use it to find overlooked connections, to validate the importance of their work and their peers’ or to assess, from a citation-based metric, declining or emerging research topics.

The conventional approach of reviewing literature, that for the purpose of this work is referred to as a ‘traditional literature review’, often relies on the expertise of scientists in the field to identify and select the key achievements to be summarized and reported (*e.g*. the EBPR book chapter by Wentzel and colleagues [5]). As an alternative or in addition, one can rely on searching a scientific publication repository, *e.g*. Clarivate Analytics’ *Web of Science* (WoS) or Elsevier’s *Scopus*, recognized as the two main databases [6], for publications with specific keywords. Then, the search might focus on existing and relevant review papers for a summary of a selection of findings, or be carried out manually facilitated by different sorting mechanisms such as date, authors, journals or further keywords. The final assessment and analysis of the content of the publications is then done manually.

In addition, for a non-expert or newcomer, and particularly when a research area is large, total citation-count-based ranking and filtering can be used based on indices such as the Science Citation Index [7]. This approach can already reduce some of the time spent on manual selection of key publications, by using total citation count as an indication of the impact and/or popularity of a certain work, it can also bias the end selection in two main ways. First, it can skew the results towards older ‘highly-cited’ publications which by definition have had more time to accumulate citations [8], to the detriment of relatively more recent ones whose contribution may be more significant in the current research context. Second, it may favour the selection of publications concerned with one specific topic, where the average citation count might be higher, obfuscating the importance of less ‘citation-popular’ ones in the same research field. The latter may occur particularly if the topics are smaller and/or newer, as could be the case with emerging fields.

To account for this, the companies behind the scientific repositories are increasingly developing new metrics and tools. For example, WoS identifies a custom group of *Highly Cited Papers*, defined as the top 1% of publications from each of the 22 *Essential Science Indicators* (ESI) research areas based on citation data from the last 10 years. While this selection is specific to both the research area and the year of publication, the resolution cannot be increased to more precisely-defined research fields or topics. For example, in the case of EBPR publications, they would be categorized as ‘Engineering’, ‘Environment/Ecology’ or ‘Microbiology’ [9] and their citation record will be compared to publications in very different fields with potentially different citation trends such as civil engineering, climate change or medical implications of bacteria, to name just a few [10]. Therefore, whilst being a useful research benchmarking tool, they may not be useful in facilitating the approach of a ‘traditional literature review’.

Another approach to review a research field would be to use citation networks. These are graph representations of scientific domains where nodes represent individual publications and the edges drawn between them represent a measure of their relatedness [11]. Closely related publications form clusters from which key topics can be identified. Such networks have recently been used to study the flow of information within and between disciplines [12], to discover the core items in a given research area, as well as to group similar ideas, findings and/or methodologies [13]. Maps have been produced at the level of institutions, journals and authors, as well as publications and their keywords in order to examine specific research themes, their geographical distribution, citation or communication patterns among researchers [14, 15]. Different similarity measures have been developed based on bibliographic coupling (BC), co-citation (CC), direct citation and other hybrid citation approaches [13]. The ability of these approaches to accurately identify coherent and independent topical clusters has been validated by survey-based verification of the resultant clusters [16, 17]. However, whilst extremely useful to distinguish between topics within a research area and view their size and their linkages, citation networks alone might not deliver all the desired information in a literature review. Additional temporal information provided by specific benchmarks to select key publications and tools to analyze citation trends over time [18] would be needed to determine areas of particular interest and their emergence or decline in popularity. Another important aspect concerning the use of citation networks to facilitate literature reviews is that the choice of which methodology to use to produce a citation network (*e.g*., BC vs. CC) might not be easily accessible to scientists unfamiliar with them. Therefore, the potential benefit of using such techniques in reducing manual labour might be offset by the time needed to surpass the bibliometric learning curve.

Therefore, with the overall aim of facilitating and automating the analysis of a research area to produce a literature review, especially for non-bibliometric specialists, this investigation presented two distinct but complementary objectives. First, in section 2, using the EBPR research area as an example, we combine, develop and apply different bibliometric techniques to suggest a framework that automates the article selection in literature reviews, while providing additional information regarding the key areas in a certain research field and their trends over time. Second, in section 3, we demonstrate the framework’s utility by applying it to the research domain of EBPR, as available via *WoS*, to produce a bibliometric analysis and a review of its key developments in the past 40 years (1975 to 2017). With the increase in publication rates in all fields [19], the results from this work provide a combination of tools that can facilitate the process of a “traditional literature review” and make a contribution to the way that increasingly larger pools of information can be processed and curated.

## 2 Bibliometric framework for automated article selection in a literature review

### 2.1 Dataset of the EBPR research domain

All data was retrieved from *Clarivate Analytics’ Web of Science* (WoS). Only documents classified as published articles, proceedings papers and review papers were taken into consideration to reflect sources for which citation indices are available and well-established. The range of published material was limited to the years between and including 1975 and 2017. Selection of the lower bound was based on *a priori* knowledge of the date of inception of EBPR as a distinct phenomenon [2]. The search gathered the union of all articles found via the terms “enhanced biological phosph* removal”, “biological phosph* removal”, “biological nutrient removal”, “EBPR” and “BNR”. To minimize the presence of unrelated articles, this list was filtered by the intersection with those found by the term “phosph*”. The full search query specific to *WoS* was: TS = ((“enhanced biological phosph* removal” OR “biological phosph* removal” OR “biological nutrient removal” OR “EBPR” OR “BNR”) AND “phosph*”) AND PY = 1975-2017. The ‘full record and cited references’ was assembled and downloaded on the 1st of September 2017. Analysis of the resultant corpus was conducted using *Python* and the *Circos* visualization tool [20]. The final dataset consisted of 2671 articles, reviews and conference proceedings.

### 2.2 Citation approaches

**Fig 1:**
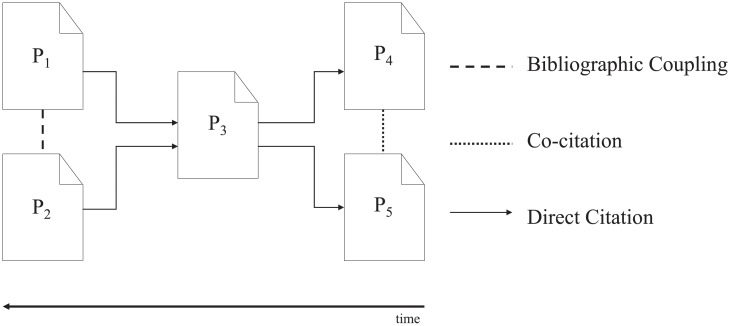
Difference between bibliographic coupling and co-citation. Bibliographic coupling links papers P_1_ and P_2_ that cite a common reference P_3_. Co-citation links papers P_4_ and P_5_ that both appear in the reference list of P_3_.

As there are different ways to construct bibliographic citation networks, two methods of association were compared based on their ability to isolate meaningful and cohesive clusters using the record of cited references within the EBPR corpus: *bibliographic coupling* (BC) and *co-citation* (CC) [21]. Both methods consider that one node is equivalent to one publication, nodes are connected to each other by *edges*, the *degree* of a node is the number of edges connecting it to all other nodes in the network and the strength of each edge is determined by the number of edges between two nodes. As illustrated by P_1_ and P_2_ in Fig 1, two nodes are connected by a BC edge if they share a common reference within their respective reference lists. On the other hand, two nodes are connected by a CC edge if they are both located on a common reference list of a third publication, as illustrated by P_4_ and P_5_. In each case, it was assumed that nodes possessing a high degree represent key ideas, methodologies and/or scientific findings in a particular research domain and that strong connections to these nodes form one particular research topic. The analysis of this distribution can hence be used to partition items within the EBPR corpus into distinct topics. The clustering itself was performed using *VOSviewer ver. 1.6.6* [22].

### 2.3 Criteria for the comparison of citation approaches

The main properties of interest were (a) the number and respective size of the resultant clusters, (b) the size of the largest connected component, (c) the internal coherence of nodes in each cluster and (d) the external isolation of nodes within a given cluster from all others. To determine these, the cluster size was normalized by dividing by the number of different clusters in the corresponding citation network. The edge strength between nodes was normalized to correct the data for differences in the number of nodes in the resultant citation networks, as shown in Eq 1 [22]:

$$\begin{array}{c} L_{\text{norm}}\left(i,j\right)=\frac{2\;c_{\text{total}}}{c_{i}\;c_{j}}\;c_{ij} \end{array}$$ (1)

where *c*_*i*,*j*_ is the number of edges between nodes *i* and *j*, *c*_*i*_ (*c*_*j*_) is the number of edges connecting to node *i* (*j*), and *c*_total_ is the total number of edges in the network. Specifically:

$$\begin{array}{c} c_{i}=\sum_{j\neq i}c_{ij}\;\text{and}\;c_{\text{total}}=\frac{1}{2}\sum_{i}c_{i} \end{array}$$ (2)

#### Degree of internal coherence

This criterion depends on two factors: i) the intra-cluster edge strength and ii) the intra-cluster density. The intra-cluster edge strength was calculated for pair-wise nodes within cluster *C* according to Eq 3 [23]:

$$\begin{array}{c} S_{\text{intra}}\left(C\right)=\frac{\sum_{i}\sum_{j}L_{\text{norm}}\left(i,j\right)}{\left(\begin{array}{c} n\\ 2 \end{array}\right)} \end{array}$$ (3)

where *n* is the number of nodes in cluster *C* and *L*_norm_(*i*, *j*) is the normalized strength of the edge between nodes *i* and *j*, provided that {*i*, *j*} ∈ *C*.

The intra-cluster density was calculated as the ratio of all existing edges between nodes belonging to the same cluster, *E*_intra_(*C*), to the maximum possible number of edges, as given in Eq 4:

$$\begin{array}{c} D_{\text{intra}}\left(C\right)=\frac{2E_{\text{intra}}\left(C\right)}{\left(\begin{array}{c} n\\ 2 \end{array}\right)} \end{array}$$ (4)

#### Degree of external isolation

This criterion depends on two factors: i) inter-cluster edge strength and ii) the inter-cluster density. The inter-cluster edge strength was calculated according to Eq 5 [23]:

$$\begin{array}{c} S_{\text{inter}}\left(C_{i},C_{j}\right)=\frac{\sum_{i}^{n}\sum_{j}^{m}L_{\text{norm}}\left(i,j\right)}{n\;m} \end{array}$$ (5)

where *L*_norm_(*i*, *j*) is the normalized link strength between nodes *i*, member of *C*_*i*_, and *j*, member of *C*_*j*_, where clusters *C*_*i*_ and *C*_*j*_ are comprised of *n* and *m* nodes respectively.

The inter-cluster density is given in Eq 6 [24]:

$$\begin{array}{c} D_{\text{inter}}=\frac{E_{\text{intra}}\left(C\right)}{E_{\text{inter}}\left(C\right)} \end{array}$$ (6)

where *E*_intra_(*C*) is the number of edges shared between any two members of cluster *C*, and *E*_inter_(*C*) is the number of edges connecting nodes within cluster *C* to all other nodes. Effectively, it is the inter-connectivity within a given cluster normalized by the degree of inter-connectivity between it and other clusters in the network.

### 2.4 Labeling topics in the EBPR research domain

The topic of each cluster was defined by performing a keyword analysis. For each cluster, all terms from the title, abstract and keyword fields were collected. Collections of terms used to label the resultant clusters needed to be both relevant and specific to the underlying concepts, methods and/or findings which tied its publications together. Terms were ranked according to their *term frequency—inverse document frequency* (TF-IDF) score [25], as given in Eq 7. The formula was adjusted to take into account not only the distribution of a term among different publications in the corpus, but among different clusters as well. For a given term ‘*i*′:

$$\begin{array}{c} TF-IDF\left(i\right)=\frac{f(i,j)}{n(j)}\log\frac{N}{n_{c}\left(i\right)} \end{array}$$ (7)

*f*(*i*, *j*) is the frequency of its appearance in cluster ‘*j*’, *n*(*j*) is the number of terms which appear in cluster ‘*j*’, *N* is the number of distinct clusters in the network and *n*_*c*_(*i*) is the number of clusters in which term ‘*i*’ can be found.

### 2.5 Categorization of citation profiles

**Fig 2:**
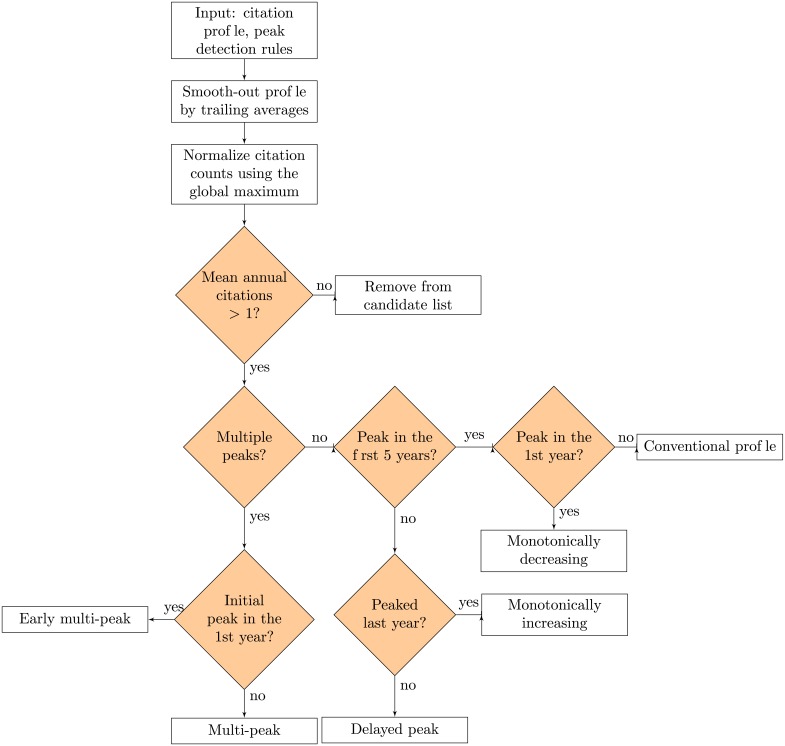
Logic tree for the citation-profile-based classification of scientific publications.

The importance of a specific publication is often measured by the total citation count. However, its citation profile, *i.e*., the historical trend of its accumulated citations, can provide an additional indication of the relevance of the paper and its interest over time [18]. This investigation considered only those papers for which a minimum of 7 years of citation history was available, having received on average at least 1 citation per year. The starting point for the characterization and classification of citation profiles was based on earlier contributions [18, 26]. First, the historical citation data was smoothed using trailing averages with moving windows of 3 years. Next, the values were normalized between 0 and 1 by dividing them by the global maximum of the time-series. Lastly, the resultant citation profile was analyzed using a local peak detection algorithm [27] in *Python* version 3.5. Two rules were defined for the identification of local maxima: (1) they must account for at least 70% of the profile’s global maximum; (2) they must be separated by more than two years. Based on these rules, publications were classified into six categories depending on the number and temporal position of peaks in their respective citation profiles. The classification procedure and definitions of the different categories of citation profiles are portrayed in Fig 2. The criteria for defining the ‘conventional’ profile were based on a prior analysis of the time-distribution of peaks in all citation profiles to determine the most common trend in EBPR publications (data shown in S1 Fig in the supplementary materials).

1. **Conventional**: Initial exponential rise in the number of citations, peaking within the first five years, followed by a decline.
2. **Multi-peak**: Several local maxima.
3. **Early multi-peak**: Initial peak within the first year of publication, followed by at least one additional peak.
4. **Delayed peak**: A single global peak only after the first five, but not in the most recent year of publication.
5. **Monotonic rise**: Increasing number of citations with a maximum occurring in the last year.
6. **Monotonic fall**: Global maximum within the first year, followed by a decline.

### 2.6 Comparing citation networks

The purpose of using citation networks in this framework is to cluster all publications into separate, well-defined and coherent sub-topics of research in a particular field. In this work, we compared two citation approaches, BC and CC as applied to EBPR. The comparison was based on their ability to deliver clusters of publication that are containing closely related (as determined by a high degree of internal coherence) and well-defined and independent from each other (as determined by a low degree of external isolation) [23, 28].

The descriptive statistics of the networks obtained with each of the citation approaches, BC and CC, are compared in Table 1. Both methods deliver the same number of clusters. However, there is a difference in the largest connected components (LCC), at nearly 25%, and in the number of edges shared between their constituent nodes, where those in the BC network shared almost four times the number of edges than their counterparts in the case of CC. In accordance with Fig 1, this suggests that publications in the EBPR research domain were more likely to be cited by common items rather than for them to cite such items themselves. As such, the BC network was expected to exhibit a significantly higher intra-cluster density. Differences in the LCC as well as median dates of publication indicate that the intersection between the entire corpus and nodes included in the LCC varied depending on the edge definition between nodes. Further inspection revealed that variation in cluster size was significant in both cases. As such, edge density was expected to vary from one cluster to another.

**Table 1:** Descriptive statistics of the BC and CC citation networks.

	Bibliographic Coupling (BC)	Co-citation (CC)	Difference (%)
Largest Connected Component	2262	1763	24.8
Number of edges	485778	123264	119.0
Number of clusters	12	12	0
Mean norm. cluster size	15.7	12.2	25
Median year of publication	2007	2005	0.1

#### Degree of internal coherence

Given that citation links are likely to happen when publications address a similar topic, this can serve as a proxy for how topically cohesive that cluster is [13]. Ideally, in a cohesive cluster, all publications (nodes) would be interconnected (high edge density) and there would be more than one connection between the same publications (high strength). As defined in 2.3, the *intra-cluster edge strength* and the *intra-cluster density* serve to quantify different aspects of the extent of internal coherence, where a coherent cluster would have the highest strength and density possible [23]. A cluster can exhibit a high intra-cluster edge strength, yet at the same time a low intra-cluster density, or *vice-versa*. For instance, in a situation where the majority of significant connections are concentrated among a limited number of publications, only a few nodes of a cluster would share strong links while the remainder of all possible node pairs would share no edges. Conversely, a large proportion of all possible node pairs may be densely interlinked with edges yet the strength of these edges could be relatively low. In this way, they are complementary and cannot be substituted for one another.

As shown in Table 2, clusters in the BC network exhibited a greater extent of mutual reciprocity among their constituent nodes, where the intra-cluster density exceeded that of CC by 49%. While the standard deviation of the intra-cluster density of BC network was comparable to that of CC, a wide variation between clusters in each network was found. This was further supported by the minimum and maximum values for each network, with those in the BC network greater in both cases.

**Table 2:** Summary statistics for the degree of internal coherence.

		Bibliographic Coupling (BC)	Co-citation (CC)
Intra-cluster density	mean	0.452	0.274
	median	0.312	0.205
	standard deviation	0.254	0.244
	min	0.171	0.0833
	max	0.867	0.489
Intra-cluster edge strength	mean	0.120	0.00945
	median	0.0119	0.00821
	standard deviation	0.210	0.00414
	min	0.0022	0.00439
	max	0.643	0.0208

This discrepancy was found to be even more pronounced with regards to the intra-cluster edge strength. As such, nodes in the BC network shared more and stronger edges with other nodes in the same cluster than with nodes from different clusters. In other words, clustering of the BC citation network was more topically-coherent than that of CC in the case of the EBPR research field.

#### Degree of external isolation

In addition to being topically cohesive, clusters should be well-separated from each other. Ideally the strength and the number of connections between publications in separate clusters should be as low as possible. This is determined by the degree of external isolation which, as defined in 2.3, can be quantified by the inter-cluster edge strength as well as the inter-cluster density.

As shown in Table 3, the mean inter-cluster density was greater in the BC network, accounting for a percent difference of about 72% compared to CC. Thus, the nodes in the BC network exhibited a higher degree of connectivity both within the same as well as between disparate clusters. In contrast, while the intra-cluster edge strength of nodes in the BC network was on average much greater than that of their CC counterparts, the edge strength between disparate clusters was not so pronounced, as shown in Table 3, with the CC network displaying a lower strength than BC. However, the percentage difference between the intra- and inter-cluster edge strengths were very different, approximately 171% and 28% respectively for BC and CC, indicating that even if CC displayed an overall higher degree of external isolation, the difference between the degree of internal coherence and external isolation is much more pronounced for BC. This was thought to be a consequence of the marked difference between the number of edges in the respective networks, in view of the relatively comparable number of nodes that shared them. Thus, although the extent of cluster-to-cluster delineation was lower in the BC than the CC network, the former was concluded to be more topically cohesive on account of scoring significantly better among the coherence criteria.

**Table 3:** Summary statistics for the degree of external isolation.

		Bibliographic coupling (BC)	Co-citation (CC)
Inter-cluster density	mean	0.0903	0.0434
	median	0.0765	0.0411
	standard deviation	0.0733	0.0152
	min	0.00560	0.0198
	max	0.222	0.0696
Inter-cluster edge strength	mean	0.00532	0.00401
	median	0.00460	0.00259
	standard deviation	0.00330	0.00346
	min	0.000460	0.000820
	max	0.0154	0.0199

### 2.7 Characterization of citation profiles in the EBPR domain

The citation profiles of publications within each cluster were re-traced in order to determine their overall temporal evolution. Specifically, differences in the composition of each cluster’s citation profiles were used as an indicator of the relevance of a particular research niche within EBPR over time. For instance, two clusters may have similar proportions of “highly-cited publications”, but if the composition of citation profiles in one was dominated by the conventional type, whereas the other leaned more towards multi-peak or monotonically increasing, then it would be plausible to assume that the former contains papers covering popular, yet mostly resolved aspects, whilst the latter comprised papers concerned with issues more relevant over time including the present.

Publications in the EBPR domain exhibited a variety of different citation profiles, based on the number and position of localized peaks. The distribution of citation profiles in different clusters is shown in Table 4.

**Table 4:** Composition of the three most frequent citation profiles, conventional, multi-peak and delayed peak profiles, as a fraction of papers identified in each cluster. Clusters I, J, K and L did not have a sufficient number of qualifying candidates with which to assess the citation profile composition.

Cluster	% of Cluster		
	Conventional	Multi-peak	Delayed peak
A	26.5	37.8	35.7
B	37.5	35.9	23.4
C	65.7	15.7	15.7
D	50.5	27.5	17.6
E	64.7	23.5	11.8
F	72.2	11.1	16.7
G	50.0	50.0	0.0
H	55.6	11.1	22.2

Although a diverse range of citation profiles was detected, on average, those of conventional, multi-peak and delayed peak were the most common, constituting 46.7%, 28.4% and 22.3% of papers respectively. The early multi-peak, monotonic rise and fall categories collectively comprised less than 3% of papers in the dataset. This suggests it has generally required at least 5 years after publication for interest in the work to reach its zenith. Based on the proportion of papers characterized by multi-peak profiles, in more than a quarter of cases, the findings of papers in the EBPR corpus sustained interest over time.

**Fig 3:**
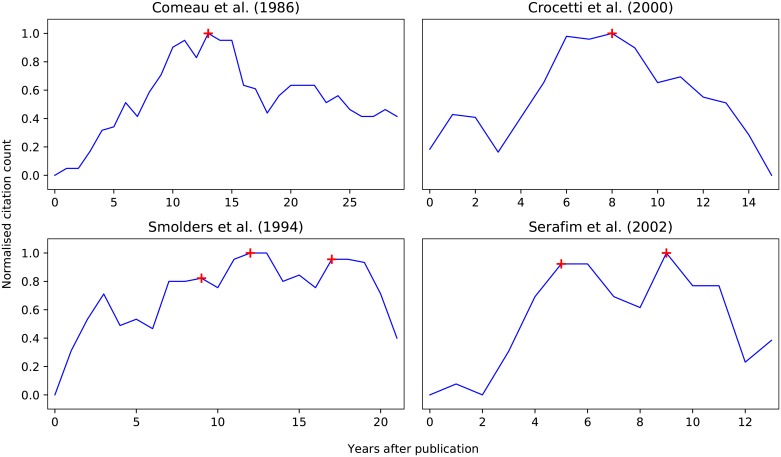
Examples of delayed-peak (top row) and multi-peak (bottom row) citation profiles.

To illustrate these citation profiles, examples of delayed peaks and multi-peaks are given in Fig 3. The first delayed peak example is the work of Comeau et al. (1986) [29]. This was among the most highly-cited papers in the EBPR corpus and is one of the most fundamental works that proposed the metabolism of Polyphosphate Accumulating Organisms (PAO). It is a delayed peak, reaching its maximum approximately 12 years after publication, but has maintained a nearly constant citation count for the last 10 years—likely because it is an important introductory read for any paper concerned with metabolism of PAO, or differences between PAO and Glycogen Accumulating Organisms (GAO). The second delayed peak is the work of Crocetti et al. (2000) [30]. It is a method paper detailing the design of 16S rRNA fluorescence probes to detect and quantify the abundance of PAO. The paper had an initial period of increase that lasted 8 years, and has been declining, likely due to the increased reliance on genetic sequencing techniques for microbial community identification. A first example of a multi-peak is the work by Smolders et al. (1994) [31]. This represents the first implementation of the anaerobic metabolic model of PAO, including the effect of pH on substrate uptake. Because it is generally cited by all publications concerned with metabolic models, as well as those investigating anaerobic metabolism, it has sustained different peaks since its publication date. The second example of a multi-peak is the work of Serafim et al. (2002) [32], a method paper detailing the visualization of intra-cellular polymers, that reached the initial peak after 5 years of publication and then a renewed interest 4 years later.

In terms of the distribution of citation profiles among the identified clusters, as shown in Table 4, Cluster A, and to some extent Cluster B, are the only ones in which the conventional profile does not dominate, having an almost equal proportion of multi-peak and delayed-peak profiles. Conversely, the most disparate distribution of citation profiles was observed in clusters C, E and F, where more than 64% of papers were characterized by the conventional citation profile.

#### Relationship between impact and distribution of citation profiles

**Fig 4:**
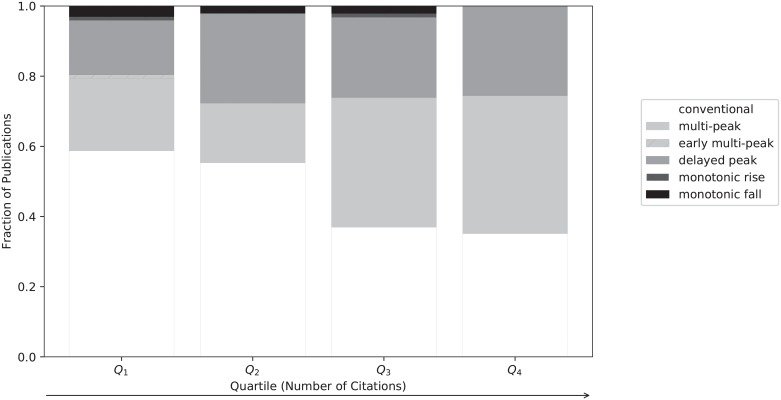
Composition of each citation quartile in terms of the six classes of citation profiles, where quartiles are determined based on the frequency of total citation counts. The arrow indicates the direction of increasing number of citations.

This work investigated whether there was a relationship between the total number of citations of highly-cited papers in the EBPR domain and their citation profiles. The corpus was segmented into citation quartiles. The composition of each quartile in terms of the six citation profiles was determined, as shown in Fig 4. The result is the conditional probability of a publication exhibiting a particular citation profile given its citation count.

As shown in Fig 4, the composition of citation profiles is dominated by the conventional, multi-peak and delayed peak categories regardless of quartile. The one relatively stable group is the delayed peak category, whose composition is nearly independent of the quartile, *i.e*., the number of accumulated citations. The share of conventional profiles decreases consistently from the lowest to the highest quartile, whereas the share of multi-peak profiles increases. This indicates that papers that garner the most citations, and likely to have had an impact in the field, tend to do so by staying relevant to on-going research questions over their lifetime, rather than accumulating a burst of citations initially upon publication. This was the case for all examples given in Fig 3.

The proportion of papers with a monotonically decreasing profile decreases from the lowest to the highest quartile. This was to be expected, as papers that have a decline in citations over their lifetime would be likely have a limited total citation count. However, the opposite was not true. The share of papers characterized by monotonically increasing profiles does not increase across different quartiles. Nevertheless, both categories form only a negligible subset of the data.

#### Influence of time on the distribution of citation profiles

**Fig 5:**
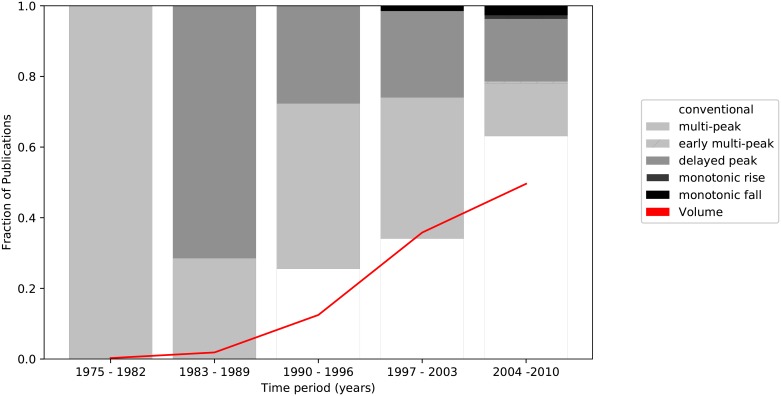
Composition of citation profiles and fraction of total publications across five evenly-spaced periods of time between 1975 and 2010. The composition of citation profiles (bars) was normalized against the number of publications in the corresponding time period, whereas the publication volume (line) was normalized with respect to the total number of publications.

The publication date would also be expected to influence the citation profiles of highly-cited publications, with older publications more likely to have been recognized for their contribution than more recent ones. As such, the likelihood of exhibiting a particular citation profile, given knowledge about the publication date, was investigated. Fig 5 shows the different citation profiles across five evenly-spaced periods of time between 1975 and 2010. Unlike Fig 4, the fraction of papers as a percentage of the total publication volume was provided, as the number of papers differs from one time period to another. The result was the conditional probability of a publication exhibiting a particular citation profile given its publication date.

As expected, the earliest bracket was dominated by the delayed-peak and multi-peak categories, whose fraction consistently decreased in subsequent time periods. Conversely, the most recent publications exhibited a predominance of conventional profiles. Viewed in the context of Fig 4, this indicates that papers in the highest citation quartile tended to have been published earlier. This result seems intuitive, as older publications, if important, would have had more time to accumulate citations gradually. With time, some of the conventional profiles in the more recent years could still shift to multi-peak or even delayed peak. However, it could also indicate that initial EBPR publications form the general foundations of the mechanisms and principles of the process and hence have maintained interest over time as reference material, whereas more recent contributions might only provide incremental knowledge or specialized information that might not be as widely relevant or applicable. Finally, it is curious to note the rise in the fraction of monotonically decreasing profiles starting from 1997, including the appearance of the monotonically increasing category in the latest time period. This can be explained by their profiles not having had sufficient time to ‘mature’, *i.e*., the impact of their contribution has not been duly accounted for. It is likely that, given sufficient time, these may shift into other citation profiles.

### 2.8 Benchmarking of total citation counts

To account for temporal bias on citation counts, a benchmark was defined where the mean number of citations per year was calculated by dividing the sum of citations received by all papers published in a particular year by their number. From now on, we will use this benchmark to define a ‘highly-cited paper’ as a paper whose citation count is higher than the mean number of citations for all publications that year.

The procedure for selecting highly-cited publications, resulting in 680 papers, proved to be less strict than the one for categorizing citation profiles, resulting in 377. These correspond to 25.3% and 14.1% of the corpus respectively. The proportion of highly cited papers agrees with the reported skewed distribution, where approximately 20% of publications accumulate 80% of citations [33].

**Fig 6:**
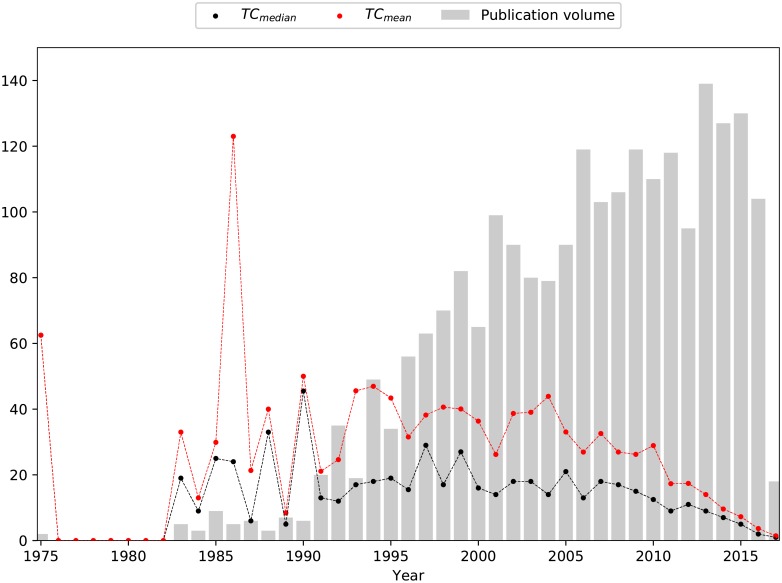
Profile of the mean and median number of citations received by papers published in a given year. Bars indicate the number of papers published in a given year.

The temporal profile of the median number of citations, as seen in Fig 6, follows that of the mean closely, albeit with a (negative) vertical offset. This was in line with expectations, as it is known that the citation frequency follows a long-tail distribution, *i.e*., Zipf or power law [17], where only a small minority of publications accumulate very high citation counts, with the vast majority of papers being cited only very sparingly. The seemingly anomalous peak in 1975, where the mean and median coincide is due to the occurrence of only a single paper that year in our dataset, the initial paper by Barnard et al [2]. The other peak in 1986 arises due to the importance of contributions from Wentzel et al. and Comeau et al. [29, 34], which have been widely cited ever since, owing to the on-going debate concerning the biochemical pathways of the organisms responsible for luxury phosphate uptake.

The general trend is a rising number of citations from the initial to the middle period of the lifespan of the EBPR domain, after which the average number of citations declines. This signified that key contributions to the understanding of EBPR took place roughly between 1990 and 2005. The decline of the mean citation count in recent years would be expected possibly for two reasons: (1) these papers have not yet had the benefit of time to accumulate citations, *i.e*., to have had the full impact of their contributions duly recognized; (2) the diversification and specialization of topics within EBPR could lead to a reduced number of citations for each new contribution. This highlights the usefulness of benchmarking publications against their peers in the same year when selecting publications for a literature review as opposed to just considering a total citation-count that might not reveal more recent but potentially relevant contributions. An example of this, is that by using this benchmark we were able to include in the literature review in section 3.4, in Cluster D, 3 publications with different citation counts but all considered highly-cited. These were the work by van Loosdrecht et al in 1997 [35] (top 50 of total citation count) on the importance of bacterial storage polymers, that received almost 2 times as many citations as the work by Lopez-Vazquez in 2009 [36] (top 100 of total citation count) on the effects of carbon source, pH and temperature and almost 10 times as many citations as the work by Carvalheira et al in 2014 [37] (not even in top 500 of total citation count) on the impact of aeration on the competition between PAOs and GAOs.

### 2.9 Recommended workflow and discussion

Section 2 presented and discussed a combination of bibliometric techniques to objectively select articles to include in a literature review. Based on the findings as applied to EBPR, we recommend the following work-flow as the basis for applying this concept to other domains.

First, in order to determine key areas of research within the field, partition the publications into distinct, non-overlapping clusters using *VOSviewer* [22]. In this case, the citation network obtained via BC yielded more topically-coherent clusters. Readers should note that this may not be the case for their dataset of interest. However, the analysis can be run for multiple citation approaches and the most suitable one selected based on the highest intra-cluster edge density and strength, complimented by the lowest inter-cluster edge density and strength, as detailed in section 2.3 and discussed in section 2.6. The topic of each cluster can be inferred using terms extracted from the title, abstract and keyword entries ranked according to their TF-IDF score, as described in section 2.4.

Research trends and the contribution of each topic to the research field as a whole can be investigated through differences in the composition of citation profiles from one cluster to another, as detailed in Section 2.5, by distinguishing between declining, sustained or newly emerging trends, as well as by differences in the distribution of publication dates, as discussed in section 2.7 and then further in sections 3.3 and 3.4.

Finally, the articles to manually review and include in the literature review can be chosen from a list of highly-cited publications in each cluster using the benchmark described in section 2.8. This work used the mean number of citations received by papers published in a given year as the benchmark for defining highly-cited publications. For the literature review itself, as the goal was a high-level description of key events in EBPR history, in each cluster only the top highly-cited paper in each year, excluding reviews, was selected. If a review was the highest-cited paper in that year it was also included in the analysis. However, depending on the objectives of the analysis, both the benchmark definition and the article selection criteria can be adapted to more or less strict requirements.

#### Comparison of this framework with alternative techniques

In essence, a literature review consists of the (1) selection of articles for review and (2) a review of the selected material. The objective of this work is to facilitate and automate the first process, that, if done in the traditional sense, requires the author to be familiar with the subject matter, so as to boil down the whole body of literature into its most critical components. With larger fields and increasing number of publications, this task often becomes time-consuming and difficult, especially for a newcomer in the field, with little prior knowledge of the subject matter.

To illustrate the advantages of the proposed framework, Table 5, compares it to a few other options and tools available to produce and/or facilitate the review of a given field, including the ‘traditional literature review’, other bibliometric techniques such as analysis of the citation network, citation profiles, as well as an example of an existing WoS indicator for highly-cited papers.

**Table 5:** Comparison of automated features in literature review methodologies. TC stands for the ‘total citation’ count.

	This work	Traditional review	TC ranking	Network analysis	WoS Highly Cited Papers	Citation profile analysis
Identification of sub-topics	✓			✓		
Custom resolution of sub-topics	✓			✓		
Identification of highly-cited publications (overall)	✓		✓		✓	
Identification of highly-cited publications (per year)	✓				✓	
Identification of highly-cited publications (per sub-topic)	✓			✓	✓	
Assessment of sub-topic temporal trends	✓			✓		✓

Given a sufficiently large data-set, our framework offers a more time efficient and automated way of conducting the first step, *i.e*., of arriving at a manageable selection of articles that highlight the most important elements of a given field. More importantly, these can be selected by sub-topic and also by year, which may reduce biases with respect to older and more highly-cited publications or reviews, as well as to the popularity of sub-topics. Therefore coupling clustering methods with benchmarks leads to a more selective result, where the date of publication as well as the research niche are weighted in addition to the citation count.

As the selection of articles for review is based on metrics determined from the metadata of the corpus of interest, there is only a minimal prerequisite in terms of prior knowledge of the research area to be reviewed—basic familiarity of the terminology to extract it. In addition, the constituent sub-topics can be identified based on clustering methods as opposed to sifting through the literature to (1) decide on how many niches it would be sensible to divide the corpus into, and (2) allocating articles to each. For the experienced audience, the framework offers not only an alternative way of organizing the body of knowledge, but also to refine this organization into progressively more specific niches by re-running the clustering analysis on a smaller subset.

Lastly, coupling clustering methods with the analysis of citation profiles can lead to further insights into the development of the field over time. Namely, differences between the distribution of different citation profiles among the identified clusters may indicate the importance of different sub-topics at various points in time, allowing inferences about the future direction of the field to be made.

#### Limitations of the applied methods

It must be acknowledged that the results presented in this work depended on the quality of the input metadata. The first source of bias, often referred in bibliometric literature, is that this work only considered papers written in English. While this was unavoidable, due to the background of the authors and the origins of the EBPR domain, research articles in other languages could be considered in future studies provided that citation information is available across the data sets in question. However, this might be more of a problem when comparing the scientific productivity at national level [38] rather than when analyzing a whole research field. Even if some distortion could result from this bias, since this framework deals essentially with highly-cited publications, it would only occur if a significant portion of the leading researchers in the area were publishing in languages other than English.

Further, it is expected for there to be an interaction between the paper type and the citation approach, *e.g*., the connections with review papers are stronger by definition (see section 2.2) in the case of CC than BC. This could have resulted in more than one cluster concerned with the same topic, because different review papers can be expected to cover the same subject matter but at different stages in time.

There might also exist a bias related to the nature of each paper, *e.g*., papers introducing a novel method, but which may not have contributed to the development of novel theories in the field. However, whether the impact of a paper (represented by the citation count) should be gauged by direct or indirect means remains a matter of debate. The main effect of this bias would manifest in the identification of highly-cited publications, *e.g*., in the automated selection of articles for a more in-depth review, as opposed to allocation of publications during clustering, therefore the bias could eventually be circumvented by manual assessment.

Another important point is that citation-based review approaches assume that the many experts in the field cite new papers based on the recognition of their importance instead of themselves using citations as a proxy for importance. This could negatively bias the attention paid to newer works.

Lastly, this work did not account for the effect of self-citations. While this would have served to inflate the citation counts of certain publications, whether this effect was mainly artificial, contributing to the distortion in the cluster analysis, or a natural consequence of formal communication in the EBPR domain can only be determined on a case-by-case basis.

One potential way to account for the myriad of reasons one paper cites another, and thereby account for the aforementioned biases, would be to pinpoint the location of the reference within the cited paper. Papers cited mainly in the ‘introduction’ (background theory, historically important work) could be expected to be different from those cited in the ‘materials & methods’ (techniques) or ‘discussion’ (recent papers of similar nature) sections. Different weightings could then be applied based on the specific type of review and/or analysis most appropriate to the research question at hand. While we acknowledge that this would have provided an additional dimension to the results, the required information was not part of the WoS metadata. A more comprehensive data collection step would be required, followed by data mining of the underlying papers themselves, rather than the metadata.

The bibliometric analysis and literature review obtained using the framework developed in this work is presented in section 3.

## 3 EBPR: Bibliometric analysis and literature review using automated article selection

The bibliometric framework described in section 2 was applied to the EBPR corpus in order to provide a bibliometric and systematic review of the field. The goal for this approach was to understand, after 40 years of research: 1) the key topics within the field; 2) infer their importance and development through their size and inter-relationships; 3) analyze their temporal evolution as determined by citation-based indicators; 4) review each topic’s key research developments as determined by an automated citation-based selection.

### 3.1 Identification of the different topics in EBPR research

Twelve clusters, each corresponding to one main EBPR research topic, were identified. Their topic, as determined from keyword analysis (see section 2.4) on the set of highly-cited publications, size and median publication year are presented in Table 6. Clusters A, B, C and D account for 68% of all publications. Furthermore, most of the highly-cited and review papers (Table 7) are also concentrated in these clusters, notably in cluster C.

**Table 6:** TF-IDF based decomposition of research problems in the domain of EBPR, ordered by cluster size.

Cluster	Research Problem	Size	Size	Median
			(% of Corpus)	Publication Year
A	Early development of EBPR	534	23.6	1997
B	Combining EBPR with N-removal	419	18.5	2008
C	Identification of EBPR microbial communities	314	13.9	2008
D	Metabolism of EBPR organisms on mixed substrate	275	12.1	2009
E	ASM Models and MBR Applied to EBPR	225	9.9	2009
F	VFA generation from WAS and N_2_O emissions	168	7.4	2013
G	P-recovery from WAS	166	7.3	2011
H	BNR with aerobic granular sludge	101	4.5	2013
I	Characterization of AS flocs	29	1.3	2005
J	P-removal using *Acinetobacter* monocultures	15	0.7	2008
K	BNR from aquaculture wastes	13	0.6	2013
L	Application of micro-electrode sensors to AS	6	0.3	2010

**Table 7:** Characteristics of each cluster including the two most common citation profiles and number of highly-cited and review papers Clusters I, J, K and L did not have a sufficient number of qualifying candidates with which to assess the citation profile composition.

Cluster	Two most Common		Citation Profile		Highly-cited	Highly-cited	Review Papers
	Citation Profiles		(% of Cluster)			(% of Cluster)	(% of Reviews)
A	multi-peak	delayed-peak	37.8	35.8	111	20.8	18.5
B	conventional	multi-peak	37.5	35.9	99	23.6	6.7
C	conventional	delayed-peak	65.7	15.7	141	44.9	28.6
D	conventional	multi-peak	50.5	27.5	98	35.6	9.2
E	conventional	multi-peak	64.7	23.5	44	19.6	9.2
F	conventional	delayed-peak	72.2	16.7	78	46.4	5.9
G	conventional	multi-peak	50.0	50.0	57	34.3	16.0
H	conventional	delayed-peak	55.6	22.2	46	45.5	3.4
I	-	-	-	-	3	10.3	1.7
J	-	-	-	-	0	0	0
K	-	-	-	-	2	15.4	0
L	-	-	-	-	1	16.7	0.84

The range of topics in cluster A was broad, with significant overlap with clusters B, C and D. For instance, publications concerned with metabolic pathways in cluster D built upon the findings reported in cluster A to take into account the additional challenge of modelling (1) PAO together with GAO, (2) the uptake of different carbon sources and (3) the differentiation of poly-*β*-hydroxyalkanoates (PHA) monomers, among other aspects. Similarly, while publications focusing on the isolation of PAO in cluster A relied primarily on culture-dependent techniques, those in cluster C employed primarily culture-independent methods to elucidate both the identity as well as function of the organisms in question. Thus, cluster A was an amalgamation of publications which constitute the early development of the EBPR domain.

Publications in cluster B further expanded on the topic of simultaneous nitrogen (N) and P removal that commenced in cluster A. Publications in Cluster C focused on the identification and genetic characterization of PAO and GAO. The timeline of the cluster demonstrates how initial efforts were concerned with finding single organisms involved in EBPR, whereas current efforts increasingly focus on whole systems-biology analyses beyond EBPR systems to consider the wider AS ecosystem. Although it is dominated by publications with a conventional citation profile, it remains, as shown in Table 7, one of the areas with the highest impact, containing the highest number of highly-cited publications (as well as the highest number of reviews), indicating that this has been the central research question throughout the lifetime of the EBPR domain.

Publications allocated into cluster D cover two closely related and inter-dependent topics: (1) the development of metabolic models that predict the behaviour of EBPR organism on multiple substrates, and (2) the characterization of their PHA production capacity. This information has been extensively used to determine the factors affecting competition between PAO and GAO. Clusters E to H deal with more specific topics within the EBPR domain, constituting approximately 29% of all publications. Such topics include mostly variations of the conventional EBPR configurations, *e.g*. membrane bio-reactors (MBR) and granular sludge, as well as process enhancements, *e.g*. volatile fatty acid (VFA) production and P-recovery from waste activated sludge (WAS). Finally, clusters I, J, K and L account for less than 3% of the EBPR domain, indicating niche areas of research.

### 3.2 Historic development of EBPR topics

**Fig 7:**
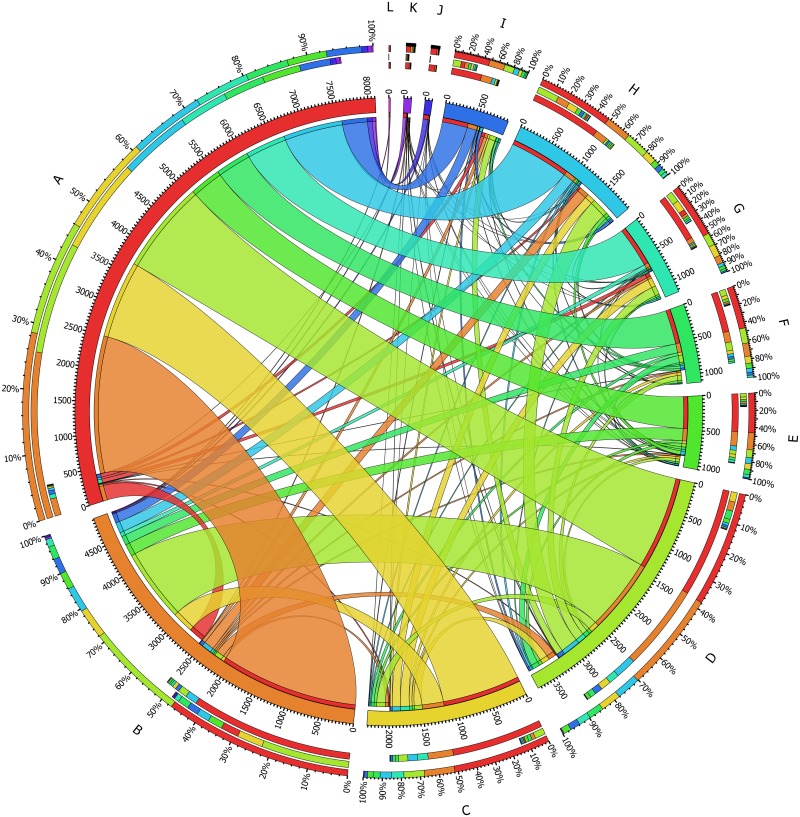
Chord diagram of the flow of citations between the identified clusters, excluding intra-cluster citations. Outer-most arch: sum of all links to and from a given cluster. Second outer-most arch: inward links, representing citations received from publications in other clusters. Third outer-most arch: outward links, representing citations given to publications in other clusters.

Fig 7 presents the different clusters along with the citations that link them together.

From Fig 7, patterns in the flux of knowledge can be determined based on the fraction of inward and outward citations, *i.e*., the number of citations a given cluster received, and the ones that it gave to others. If the number of inward citations is greater than the number of outward ones, then this cluster can be expected to form a reference basis for other topics in the EBPR domain. This is the case for cluster A, where the ratio of inward to outward edges was not only the highest, at 18.9, but also the only one to exceed a value greater than 1. In other words, publications in cluster A were the only ones to garner more citations from those in other clusters than it reciprocated. This was within expectations, as publications in cluster A were the oldest, with half being published before 1997. It is clear from Fig 7 that cluster A is the target of the largest fraction of outward edges from all other clusters, suggesting that it forms the theoretical and/or empirical foundation for EBPR. For example, the works of Comeau et al in 1986 [29] and Smolders et al in 1994 [31] are largely recognized as the foundations for the biochemical models of EBPR, whereas the work of Kuba et al in 1993 [39] and the work of Cech and Hartman in 1990 [40] set the first steps for denitrification in EBPR and the competition by GAOs, respectively. Further examples can be found in section 3.4.

In the case of cluster B, which deals predominantly with anoxic EBPR, the number of inward citations was approximately equal to the number of outward ones, indicated by a ratio of 0.94. From Fig 7, it is clear that most of the citations given by cluster B are directed to publications in cluster A (approximately 80%), whereas the majority of the citations it received originate from publications in cluster D. This suggests that cluster B built on the foundations laid down by A, whilst simultaneously influencing cluster D, associated with the development of metabolic models for EBPR organisms on mixed substrates. The ratio of inward to outward citations in clusters C, D, E, and F were low, averaging at 0.18, under strong influence from clusters A and B. This suggests that these topics, although making use of EBPR principles, did not further advance the core knowledge of the process itself, branching instead into other areas.

Despite their smaller size, clusters G and H had a higher fraction of inward to outward citations than C, D, E and F, at 0.58 and 0.62 respectively, suggesting that they have had more wide-reaching influence on other topics in EBPR. This makes intuitive sense for cluster H, as findings in the study of aerobic granular sludge can be expected to have made important contributions elucidating metabolic processes, spacial distribution and identity of microorganisms relevant to EBPR, as confirmed in Fig 7 by outward links to clusters D, B and C respectively. As for cluster G, it is possible that the high fraction of review papers, shown in Table 7, was responsible for the difference in the ratio of inward to outward citation, as they tend to gather more citations than ordinary papers. Alternatively, the high ratio of inward to outward edges may be explained by the fact that the associated topic, P-recovery from WAS, provided an additional justification for EBPR.

### 3.3 Identification of temporal trends in EBPR research

**Fig 8:**
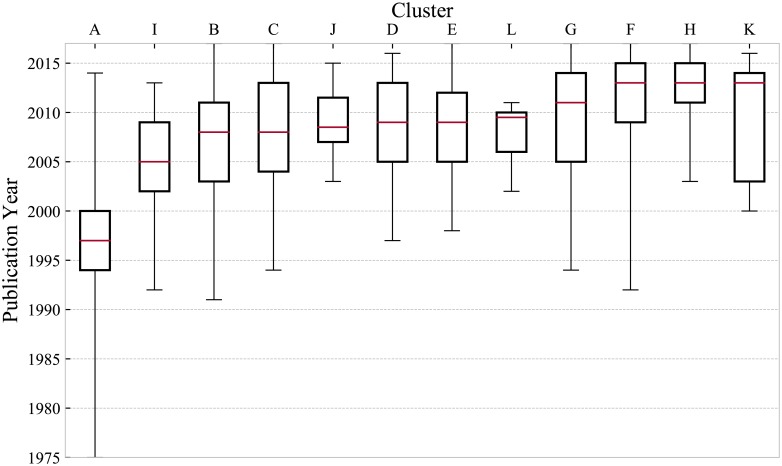
Boxplot of the publication year distribution for each cluster, indicating the median (band), interquartile range (box), as well as the minimum and maximum (whiskers) values. The inter-quartile range indicates the time period during which 50% of papers in a cluster were published.

Fig 8 shows the distribution of publications over time in each cluster ranked in order of the ‘oldest’ to ‘youngest’ moving from left to right, based on the median year of publication. In addition, the two dominant citation profiles, as well as the number of highly-cited publications are shown in Table 7.

Out of the four core EBPR clusters, A is, as expected, distinctively the oldest. It covers the widest range of publication years, however it is in decline as the value of the 75^th^ percentile occurred nearly two decades ago in 2000, and the last publication occurred in 2014. It is the only cluster for which the most common citation profiles are multi-peak and delayed peak, suggesting that the majority of the papers in this cluster have either sustained interest over different periods of time or reached their peak number of citations at least 5 years after publication.

Clusters B, C and D seem to have all developed at the same time, with an interval of roughly 10 years after cluster A, all characterized by the delayed-peak or conventional as the most common citation profiles. Clusters B and D seem to be slightly in decline, although the former still contains recent publications, likely in relation to the on-going debate of anoxic pathways of PAO and GAO.

It was surprising that the most common citation profile by far in cluster C was the conventional type, since genetic profiling of microbial communities is currently an area of increasing research intensity, owing to the recent developments in genomic, proteomic and related techniques. This may have been due to the fact that publications using such techniques were clustered in line with the particular ecosystem of interest, *i.e*., split between microbial communities specific to conventional suspended growth processes in cluster C, those specific to membrane-assisted configurations in cluster E and those specific to aerobic granular sludge processes in cluster H. Nevertheless, cluster C housed both the absolute number as well as the highest fraction of highly-cited publications and review papers respectively.

Clusters E, F and H developed more recently, with median publication years lying between 2009 and 2013. Cluster F, associated with VFA production from WAS, was the first to be addressed, with initial publications in 1992, whereas cluster H, associated with granular sludge, was the most recent technological development, with initial publications occurring a decade later.

All of the smaller clusters (I to L) seemed to have well defined lifespans, *i.e*., where the most recent publication year occurred just before the time of this study. Clusters I and J are the oldest and seem to be in decline, as expected, since they relate to very specific topics with limited scope for expansion. Cluster K, associated with the application of Biological Nutrient Removal (BNR) to aquatic wastes, is still relatively new, with potential for further development. Conversely, cluster L, associated with the development of micro-electrode sensors, is very well defined in time, from 2002 to 2011, with the median biased strongly towards the more recent end of the cluster’s lifespan.

### 3.4 Review of the key developments of 40 years of EBPR

The following sub-sections present a review of EBPR research for clusters obtained in Table 6. Only key highly-cited publications were selected, one per publication year in the lifespan of each cluster, in accordance with section 2.9.

#### Cluster A: Early development of the EBPR research domain

Publications in cluster A were concerned with elucidating the biochemical mechanism of EBPR, particularly at a time where molecular methods to identify and study PAO *in situ* were not available.

EBPR, much like the AS process, was first discovered by chance. Due to increasingly stringent requirements for the removal of organic matter and N and its resultant increased economic burden due to aeration, research shifted from aerobic to anaerobic treatment. In addition to low nitrate, the concentration of P in the effluent could be consistently ensured at levels below 1 mg/l without the addition of chemical precipitants [2]. Full-scale EBPR plants had been built at a time when the underlying mechanism of EBPR was still unknown. Therefore the first steps focused on defining operational aspects such as the influence of the concentration of organic pollutants, nitrates and dissolved oxygen (DO) on the efficiency of P-removal [41, 42]. The introduction of the first theoretical frameworks occurred in 1986 [29].

Poly-phosphate is thought to serve as the sole source of energy for the anaerobic uptake and storage of organic substrates in the form of PHA, alongside reducing equivalents obtained from the tricarboxylic acid (TCA) cycle [29, 43]. In aerobic conditions, stored poly-*β*-hydroxybutyrate (PHB) would be expended for growth and P-uptake for the replenishment of poly-phosphate reserves. The synthesis and degradation of PHA and poly-P were regulated by the ratio of NAD/NADH and ATP/ADP respectively, themselves dependent on the intra/extra-cellular balance of substrates and the availability of electron acceptors. An alternative model diverged in the source of reducing equivalents for PHA formation, as well as in the mechanisms for the uptake of organic substrates, where glycogen was the source of energy and reducing power and transport was active rather than passive [44].

Reliance on both the TCA cycle and glycolysis to resolve the energy requirements for substrate uptake and PHA synthesis has been suggested [44, 45] to explain the varying composition of the synthesized PHA [43, 46], as well as to balance the internal redox potential. Despite initial findings from NMR tracer experiments indicating that the anaerobic degradation of glycogen proceeded through the Entner-Doudoroff (ED) pathway, the possibility of utilizing the Embden-Meyerhof-Parnas (EMP) pathway remained in question. The last difference between the two models was the mechanism of organic substrate uptake where, through the variation of anaerobic P-release under different pH, it was demonstrated that an active means of transport was in play [31]. Nevertheless, the supply of reducing equivalents, the pathways for glycogen degradation and the mechanism of organic substrate transport continue to be a subject of interest in light of new information concerning the identity as well as genomic potential of organisms relevant to EBPR. Alternative metabolic pathways for biological P-removal, *e.g*. in single-stage aerobic processes have also been reported [47].

Increasingly stringent regulations have been the main driver for the intensification of waste water treatment technologies. The possibility of using nitrate as an electron acceptor in lieu of oxygen was confirmed in lab-scale experiments [39]. Although stoichiometric and kinetic assays indicated that cell yield and energy production, and therefore P-uptake efficiency were lower with nitrate (NO_3_^−^) [39], selective enrichment of the sludge in denitrifying PAO (DPAO) circumvented the issue of incomplete denitrification in earlier systems, specially those with low organic loading such as MBR-assisted processes, detailed further in section 3.4.5. An initial metabolic model for anoxic P-uptake was proposed in 1997 [48].

The discovery that the deterioration of P-removal performance could be induced by the proliferation of what would eventually come to be known as GAO was a critical junction [49]. This highlighted the need to understand not only the relationship between the P-removal performance and the composition of the microbial community, but also of the precise identity and characteristics of organisms both beneficial and detrimental to the EBPR process [50].

Although a number of works investigated the factors influencing EBPR performance, *e.g*. linking P-removal to temperature [51], pH [52] or the concentration of Ca^2+^ and Mg^2+^ ions in the influent [53], these were conducted mostly in the absence of knowledge of PAO identity, nor of reliable means to quantify their population [54]. Based on cultures incubated with sludge from full-scale WWTP, P-removal in the presence of VFA was initially attributed to *Acinetobacter* [55]. It was not until the dawn of culture-independent molecular techniques that the relation between operational conditions and the composition of the microbial community could be explored directly, as detailed in section 3.4.3. It was shown via *fluorescence in situ hybridization* (FISH) that pure-culture methods had overestimated the importance of *Acinetobacter* in full-scale EBPR [56]. Noting differences between microbial communities with different capacities for P-removal, EBPR performance was correlated with the abundance of *Rhodocyclus*-related bacteria [57].

#### Cluster B: Simultaneous nitrification, denitrification and phosphorus removal

EBPR with N-removal is typically COD limited due to the competition between PAO and heterotrophic denitrifiers for carbon substrate. In addition to ascertaining the identity and metabolism of denitrifying PAO, the optimization of available COD use [58] and alternative N-removal processes such as partial nitrification and anammox [59] have been the major lines of research.

The initial findings established the existence of DPAO, along with the key operational features of anoxic P-removal. PAO found in pilot-scale AS plants could be sub-divided into two groups: those only capable of using oxygen, and those able to use both oxygen and nitrate as electron acceptors [60]. Consequently, P-uptake was more rapid under aerobic conditions since only a fraction of the PAO population was active in anoxic conditions. Simultaneous N and P removal with DPAO could reduce COD demand, oxygen consumption, as well as reduce sludge production by 50, 30 and 50% respectively [61]. While lower anoxic P-uptake rates could be attributed to DPAO only being a fraction of the total PAO population [60], it was later determined that this was also due to the lower efficiency of P-uptake using nitrate compared to oxygen as electron acceptors [62]. This was confirmed in a study of DPAO kinetics on NO_3_^−^ [63].

A method to quantify the fraction of DPAO by comparing P-uptake rates of anaerobic-aerobic and anaerobic-anoxic batch tests was proposed [62], which would play an important role in optimising simultaneous N and P-removal processes and in verifying model predictions, even after the emergence of molecular methods able to quantify this population directly. The combination of the metabolic model for anoxic P-removal [48] with the ASM2 model was used to successfully model simultaneous N and P removal in a full-scale WWTP [64]. It was also shown that since DPAO are enriched by minimising the ratio of aerobic to anoxic SRT, long overall SRT are required in order to ensure the establishment of autotrophic nitrifiers [65]. The importance of influent feed characterisation and empirical knowledge for model calibration was also highlighted.

Extensive research has been done to ascertain the role of nitrite (NO_2_^−^) on the efficiency of integrated N and P-removal systems. Although anoxic P-uptake can occur regardless of whether the electron acceptor present was nitrate or nitrite, the ratio of P to N-removed has been reported to increase along with the initial concentration of NO_3_^−^, whereas it decreased slightly with NO_2_^−^ [66]. Observing that the relative abundance of PAO decreased with respect to GAO following a nitrite shock event, it was suggested that NO_2_^−^ inhibits P-uptake in both aerobic and anoxic conditions, thereby stimulating GAO over PAO [67]. However, a stronger correlation between P-uptake with free nitrous acid (HNO_2_) rather than NO_2_^−^ was reported in an investigation of the effect of pH on nitrite speciation, where P-uptake was completely inhibited at a HNO_2_ concentration of 0.02 mg/l regardless of the degree of DPAO enrichment [68].

The capability of DPAO to carry-out simultaneous nitrification and denitrification (SND), *i.e*., partial oxidation of ammonium (NH_4_^+^) to NO_2_^−^, followed by direct reduction to nitrogen gas was investigated in an effort to further reduce COD requirements [69]. While NH_4_^+^ was oxidized without NO_2_^−^ nor NO_3_^−^ accumulation, low P-uptake rates indicated that it was DGAO rather than DPAO who were responsible for the observed N-removal activity. It is possible that HNO_2_ inhibits the aerobic synthesis of glycogen in GAO to a lesser extent than in PAO, allowing them to outcompete PAO for VFA. Despite the potential for further reduction of COD requirements, sludge production, as well as CO_2_ emissions from denitrification, SND in sequencing batch reactor (SBR) systems has not received much attention as a result of PAO inhibition by NO_2_^−^ and/or HNO_2_ [70]. SND via the nitrite pathway seems to be more promising in biofilm [71] and granular sludge [72, 73], where stratification of the microbial community and the associated concentration gradients prevent NO_2_^−^ inhibition of PAO activity.

While the presence of NO_3_^−^ in anaerobic conditions had been known to cause EBPR deterioration, it was unclear whether this was due to (1) the inhibitory effect of an intermediate molecule, or (2) COD limitations arising from competition for substrate with ordinary heterotrophic denitrifying bacteria. EBPR was found to be feasible even in the presence of NO_3_^−^ in the anaerobic zone [74]. ASM2d simulations with two-step nitrification and denitrification showed that rather than the NO_3_^−^ concentration, it was the availability of VFA in anaerobic conditions that determined EBPR performance. It has been suggested that NO_3_^−^ deteriorated EBPR not directly by inhibition of P-release, but indirectly by inhibiting the fermentation of carbon substrates into simpler molecules, *i.e*., VFAs. Indeed, using a combination of FISH and denaturing gradient gel electrophoresis (DGGE), it was confirmed that while denitrification in a full-scale SBR depended strongly on DPAO activity, the community composition varied greatly over time due to variations of influent composition [75]. The relationship between SBR operational stability and microbial diversity remains an evolving question. The interaction of microorganisms with their environment and different microbial groups is further discussed in section 3.4.3.

#### Cluster C: Identification and genetic profiling of EBPR microbial communities

The topic began with FISH investigations of full-scale AS processes to show how the role of the first isolated PAO, belonging to the genus *Acinetobacter*, might have been overestimated [76]. Subsequent studies have either used different techniques to isolate or enrich communities in putative PAO and GAO, and/or used 16S rRNA sequencing techniques to determine their taxonomic diversity. Constructing clone libraries of 16S rDNA fragments, a new genus and species was proposed, *Candidatus Accumulibacter phosphatis* [77], henceforth referred to as *Accumulibacter*, belonging to the *Betaproteobacteria* class. FISH probes designed for *Accumulibacter* [30] continue to be widely used to this day. A finer diversity within *Accumulibacter* PAO was demonstrated by way of the *polyphosphate kinase* 1 gene as opposed to 16S rRNA [78], with potentially distinct ecological roles. This was followed by the proposition of two new FISH probes to distinguish between *Accumulibacter* clades I and II (often referred as PAOI and PAOII) [79]. Other putative PAO have been investigated, including members of the *Gammaproteobacteria* class and *Actinobacteria* phylum, *e.g*. *Tetrasphaera* [80]. Other results have supported widespread occurrence and role of *Betaproteobacteria* and *Actinobacteria* as PAO in full-scale EBPR plants, although the latter did not take up PHA [81]. A similar result had been obtained in a study based on the use of respiratory quinones as biomarkers that demonstrated the influence of synthetic vs. real sewage on community selection, showing that *Betaproteobacteria* and *Actinobacteria* were the two of the most abundant organisms [82].

The work that first identified G-bacteria was allocated to cluster A [40] and GAO were subsequently categorized as a new genus: *Amaricoccus* [83]. However, the main GAO has been *Candidatus Competibacter phosphatis*, henceforth referred to as *Competibacter*, a member of *Betaproteobacteria*, for which FISH probes were designed by the same research group that designed the ones for *Accumulibacter* [84]. Other GAO have been suggested over the years and their abundance, alongside PAO, has been comprehensively analysed over three years in full-scale EBPR plants via FISH quantification [85]. Although *Accumulibacter* and *Tetrasphaera* PAO were shown to be abundant, no temporal variations were detected. *Competibacter* and *Defluviicoccus* GAO were also found to be occasionally abundant in certain plants with each site exhibiting specific PAO/GAO fingerprints. These findings were further expanded with genetic sequencing data on bacterial assembly and temporal dynamics [86], suggesting that these contained a core community of organisms with little seasonal variation. Advances in this area have been extensively reviewed [87, 88], highlighting the key mechanisms and biochemical pathways.

The diversity within *Accumulibacter* [78, 79] suggested that each clade would have different denitrifying capabilities, with PAOI being able to use nitrate as an electron acceptor, whereas PAOII not. These results were aligned with previous metagenomics findings where clade IIA has been shown not to have the necessary respiratory nitrate reductase to denitrify from nitrate—only from nitrite [89]. Other studies have focused on the influence of operational parameters such as dissolved oxygen levels on microbial dynamics and diversity of AS [90].

Finally a number of tools have emerged over the years that allow a much more substantial analysis of complex microbial communities and their genetic potential. The mapping of the proteome, using two-dimensional polyacrylamide gel electrophoresis, and later of the proteogenome, was demonstrated for *Accumulibacter* [91, 92]. The first metagenomic analysis of two EBPR sludge communities [89] provided insight into the genetic potential of *Accumulibacter* PAO, particularly clade IIA. These findings were further expanded in 2012 [93]. The sophistication of the new sequencing techniques have led to the emergence of other bioinformatics research questions that have shifted focus away from classic EBPR topics, *e.g*. new methodologies and software for correct analysis and assignment of metagenomic data [94, 95]. Further, the best approaches for primer selection in sequencing studies have been discussed [96].

#### Cluster D: Metabolism of EBPR organisms on mixed substrate

The main point of departure emphasized the importance of different species of PHA, as well as the complexities of PHA formation mechanisms [35]. Initially, the accumulation of storage polymers was considered important in view of improving sludge settleability [97].

Metabolic models offer a way to integrate information from various sources within a mathematical framework to describe and assess observations with respect to EBPR on a mechanistic basis. The driving force to their application has been the prediction of conditions favouring the growth of PAO over GAO [4]. Metabolic models developed separately for PAO and GAO [98] have been combined to predict the fraction of PAO to GAO based on the total acetate (HAc) uptake and glycogen consumption [99]. This demonstrated that cultures previously thought to be highly enriched in PAO without microbial characterization could have had sizable fractions of GAO. This approach was further employed to take into account the effect of temperature, pH and the composition of VFA in the feed, composed of HAc and propionate (HPr) at different ratios on the competition between PAO and GAO [36]. In particular, HPr has been suggested as an important selector for PAO over GAO [100, 101]. These models allowed for the rationalization of potential explanations for contradictory findings regarding microbial compositions in different lab-scale cultures, as well as full-scale systems. The stoichiometry and kinetics of EBPR conversions remain uncertain, due to factors such as pH on the P/HAc ratio or GAO activity.

In addition to the composition of the influent carbon source, the P/COD ratio has been one of the most thoroughly studied factors in the competition between PAO and GAO. While it is expected that high P/COD ratios select for PAO, complete elimination of GAO from the system has not been achieved with this ratio alone, and the precise effect of its manipulation on the population balance has not been quantified. Research on this front focuses on PAO selection in systems starved of carbon substrates and at low dissolved oxygen concentrations [37, 102].

In parallel to the development of metabolic models for PAO and GAO, the concept of PHA production from AS or EBPR sludge was proposed as a bio-degradable alternative to oil-based plastics [103]. Considerable effort has been devoted to the development of optimal bacterial strains as well as more efficient fermentation and recovery processes to reduce the cost, and by extension, increase the economic viability of PHA production [104]. Different strategies for PHA production from mixed cultures have been reviewed [105]. Interestingly, it was found that the fraction of stored substrate could be predicted by the ratio of specific PHB production to HAc uptake [106]. This provided the groundwork for a generalized metabolic model to predict PHB production and consumption in mixed cultures grown on HAc [107]. A critical step was the standardization of analytical methods to detect PHA [108]. The characterization of metabolic pathways for PHA production on single or mixed substrates has been a subject of great interest, further enabling the refinement of metabolic models applied in EBPR, namely with respect to the influence of the feed composition [109]. PHA production is tied closely to the competition between PAO and GAO by the focus on substrate uptake mechanisms [110].

#### Cluster E: Activated sludge models and membrane bio-reactors applied to EBPR

The first cornerstone was achieved with the publication of the ASM2d model that expanded the original ASM2 model to include the denitrification activity of PAO [111]. This model has been applied to different systems and subsequent modifications have been proposed, *e.g*. for the simplification of ASM3 [112], and later the inclusion of a chemical analysis in terms of pH and ion pairing and/or speciation [113]. A number of review papers were produced that kept track of the key developments in this area, *e.g*. that of Kovarova et al in 1998 [114]. Given the complexity of the ecosystem of interest, much-needed standardization and reference material for any mechanistic model for both AS and EBPR processes has been assembled [115, 116]. A note goes to the work of Hellweger [117] who reviewed the relevance of individual-based, also referred to as agent-based modelling.

An overall less-cited topic in this cluster but with nonetheless important contributions was the use of MBR for EBPR. The connection between the two topics likely stemmed from the application of knowledge included within the ASM2d model to MBR technology. The shortage of organic substrate for simultaneous EBPR and N-removal coupled with increasingly stringent regulations led to the development of MBR and integrated fixed-film activated sludge (IFAS) configurations [118]. The first successful MBR-assisted EBPR processes were trialled in both pre- as well as post-denitrification configurations, with the membrane replacing the final clarifier, achieving P-removal efficiencies above 90% [119]. Effluent P concentrations were maintained below 100 *μ*g/l under SRT characteristic of conventional and MBR-assisted plants, suggesting that long SRT do not necessarily inhibit EBPR mechanism [120]. Simultaneous N and P-removal has been successfully achieved in MBR systems, both in small-scale [121] and full-scale municipal WWTP [122]. MBR technologies for wastewater treatment has been systematically reviewed [123].

Finally, as with any MBR-assisted process, factors leading to fouling were investigated. Higher SRT were found to negatively affect both fouling and nutrient removal [124]. A similar influence on fouling was reported with regard to floc size distribution and amount of extra-polymeric substances (EPS) [125].

#### Cluster F: Generation of VFA from waste activated sludge

The shortage of VFA is often a limiting factor in wastewater [126]. This can be exacerbated by the competition for the substrate by organisms other than PAO, whereby supplementation of carbon sources is critical to support BNR. Although this requirement can be met by addition of synthetic VFA, a more sustainable and cost-effective means is to obtain them from the fermentation of WAS generated on-site. As such, extensive research has been conducted to optimize VFA from WAS from biological treatment.

The high-strength liquors generated by hydrothermal treatment in an effort to reduce waste sludge volumes had been considered a disadvantage. By controlling the composition of the resultant organic fractions, these products could be suitable to support BNR. 95% hydrolysis of sludge at sub-critical temperatures was achieved to produce highly concentrated liquors, where the fraction of HAc was found to be as high as 80% of the soluble COD [127]. The accumulation of HAc was found to be favourable under such conditions due to its resistance to both thermal degradation and oxidation. Although an SBR fed with substrate from hydrothermally treated sludge exhibited higher P-release as well as uptake rates, due to the fact that the soluble COD for the control reactor was also lower (152 compared to 307 mg/l), to what degree this difference in EBPR performance could be attributed to increased availability of VFA as opposed to higher influent VFA concentration was unclear [128].

In the fermentation process, hydrolysis is considered to be the rate-limiting step, influenced by temperature and pH to attain higher rates and more effective degradation of complex organic matter [129]. Contrary to previous findings where fermentation was found to be optimal at neutral pH [130], it has been reported that alkaline conditions led to greater VFA yields thanks to the inhibitory effect of high pH on the activity of methanogens, confining the anaerobic digestion (AD) process to the hydrolytic and acidogenic stages [126]. Pyrosequencing and FISH analysis confirmed that alkaline conditions increased the abundance of bacteria involved in hydrolysis and acidogenesis and decreased the abundance of methanogenic archaea [131]. Interestingly, the diversity of the resident archaea increased despite the overall reduction in population size. This was contrary to expectations, as only a few methanogenic archaea had been thought to grow at pH far from neutral.

VFA production can be enhanced with physico-chemical pre-treatment of sludge, an area of growing interest. Low-intensity methods, *e.g*. mechanical treatment, have been reported to improve degradation kinetics, whereas high-intensity methods, *e.g*. thermal hydrolysis, improve both the rate as well as the extent of degradation [132]. Pre-treatment with HNO_2_ seems to inhibit obligate methanogenic activity by changing the strictly anaerobic conditions to anoxic, as well as by enhancing the solubilisation of carbohydrates and proteins [133, 134]. In a similar manner, chemical surfactants improve the solubilisation of EPS, breaking down the AS floc matrix to release more substrate for VFA generation by fermentative bacteria [135]. Further elucidation of the effect of surfactants on acidogens, as well as the resultant VFA yields is required.

Finally, N_2_O has received considerable attention in recent years on account of being roughly 300 times more potent as a greenhouse gas (GHG) than CO_2_ [136]. The tendency of WWTP to decrease energy consumption by decreasing aeration, *e.g*. in implementing EBPR and/or anoxic denitrification may have adverse effects towards GHG emissions. Nitrous oxide has been observed to accumulate as soon as PHB becomes the growth substrate due to COD limitation [137]. However, N_2_O accumulation has been reported as due to the speciation of NO_2_^−^ into HNO_2_ at acidic pH rather than the competition between nitrous oxide reductase and nitrate reductase enzymes [138]. Nevertheless, plants achieving high levels of N-removal emit less N_2_O, indicating that no compromise is required between effluent quality and GHG emissions [136].

#### Cluster G: Phosphorus recovery from waste activated sludge

Although the timeline for peak P is contentious, it is clear that the phosphate rock deposits that remain are of lower grade and are becoming more difficult to access. It is estimated that roughly 20% of the global demand for phosphate rock could be satisfied by P-recovery from municipal waste streams alone. As such, there is growing interest in the technical and economic feasibility of large-scale P-recovery from municipal and agro-industrial waste streams [139].

Spurred by the greater availability of P in secondary sludge compared to using chemical precipitation, EBPR followed by crystallisation as struvite (magnesium ammonium phosphate) or apatite (calcium phosphate) has been concluded be the most effective means of P-recovery [140]. Most research into P-recovery has been conducted with struvite precipitation in mind, as the final product from the crystallisation of apatite is of lower value as a fertilizer and is more difficult to separate [139]. In addition, apatite crystallisation does not appear to occur to any appreciable extent in WWTP, given that the concentration of phosphate (PO_4_^3−^) and calcium ions must be at least 50 and 100 mg/l respectively [141]. In contrast, struvite precipitates spontaneously in WWTP environments, where high concentrations of soluble P and ammonium are present. Unintentional struvite precipitation has long been recorded as a nuisance, disrupting operation of WWTP by blocking valves, pipes and pumps. This has been exacerbated by the increase of the required nutrients as a result of greater degrees of nutrient removal. As such, controlled struvite crystallisation serves to (1) alleviate routine operational problems, (2) reduce sludge volumes by as much as 49% and (3) recover a useful raw material [142]. In this context, EBPR is most commonly coupled to AD, as P bound in organic matter within secondary sludge is hydrolyzed in the latter to produce a concentrated phosphate stream, thereby increasing the fraction of recoverable P.

The factors that affect P-removal via struvite precipitation using digested sludge from a full-scale EBPR plant have been investigated [143]. At alkaline pH, the PO_4_^3−^ concentration increases, while those of Mg^2+^ and NH^4+^ decrease, such that their molar ratio becomes favourable for the formation of struvite crystals. Struvite precipitation is strongly influenced by the ratio of Mg^2+^, NH_4_^+^ and PO_4_^3+^, and is inhibited by the presence of ions such as Ca^2+^, K^+^ and CO_3_^2−^. The optimal ratio of Mg:N:P appears to be 1.2:3:1 [144]. Further, the molar ratio of N to P can cause transformation of struvite to newberyite, which degrades the quality of the final product. The importance of using quantitative X-ray diffraction for quality control by determining the various crystal phases and amorphous content of recovered struvite-containing products has been highlighted [145].

Recently, novel means of P-recovery have been explored. For instance, instead of dosing magnesium salts at rapidly changing pH, electrolytic deposition using a sacrificial magnesium anode was shown to be an effective way to precipitate struvite at high purity, achieving a P-removal efficiency of 98%, without any by-products [144]. Finally, the need to expand our knowledge of Fe-P chemistry in order to improve the economic viability of P-recovery, highlighting that fungi, bacteria and plants routinely release Fe-bound P for their metabolic needs has slowly come to be recognized [146].

#### Cluster H: EBPR using aerobic granules

Due to the requirement of large settling tanks to retain biomass as well as large reactor volumes arising from low biomass concentrations, suspended growth processes cover large land footprints. Aerobic granular sludge (AGS) presents an opportunity to intensify the AS process, as granules are more dense and have considerably higher settling velocities than conventional AS flocs.

P-accumulating granules were first developed in an SBR [147]. These simultaneously released P with uptake of organic carbon substrates in anaerobic conditions and assimilated P in aerobic conditions. Investigations have focused primarily on the influence of the influent P/C ratio, DO levels and temperature on granule formation and characteristics, as well as on nutrient removal efficiencies. While the enrichment of PAO in the granules was positively correlated with the P/C ratio, high metabolic activity in the aerobic stage was incorrectly identified as a cause rather than a consequence of EBPR deterioration [147]. Recently, AGS in continuous flow was investigated to improve granule stability and their distribution in the anaerobic and aerobic stages [148].

By manipulating DO levels, the ratio of aerobic to anoxic volume was identified as the major factor influencing simultaneous nitrification, denitrification and P-removal [149]. Based on previous findings, a mathematical model was developed to describe AGS in an SBR [150], accounting for the effect of DO, T, granule diameter, sludge loading rate and cycle configuration. As expected, P-removal was dependent primarily on sludge age, as sufficiently long SRT is required to maintain slow-growing PAO biomass in the system. P-removal was later confirmed to be relatively insensitive to changes in temperature [151]. This was surprising, as PAO are thought to be outcompeted by GAO at temperatures above 20 °C.

Using FISH, granules towards the bottom of the reactor were found to be more enriched in PAO (relative to GAO) than those in the top of the reactor as a result of cell density differences (arising from the storage of poly-phosphate) and concentration gradients of substrate in the reactor [152]. P-removal efficiency could be maintained, even improved to 100%, by discharging WAS from the top of the reactor, effectively exposing GAO to a shorter SRT. This showed how granule heterogeneity provides different ecological niches. In addition to competition, an important finding was made concerning the coexistence of PAO and GAO in AGS systems. In systems where PAOII, incapable of reducing nitrate, were the dominant clade, coexistence with DGAO was possible, perhaps even necessary, as they contributed not only to denitrification but also indirectly to EBPR by supplying DPAO with suitable electron acceptors for anoxic P-uptake [153]. A cascade inhibition effect was identified in a study of AGS in highly saline conditions, where NO_2_^−^ accumulation arising from the inhibition of nitrite-oxidizing bacteria (NOB) led to the inhibition of both anoxic and aerobic P-uptake, particularly at Cl^−^ concentrations exceeding 20 g/l [154]. This resulted in the wash-out of PAO and NOB.

Interestingly, there has been some debate over the role of EPS, in terms of granule stability as well as its involvement in EBPR using AGS, given that they affect mass transfer in floccular systems. A metabolic model has been formulated in which poly-phosphate was hydrolyzed and synthesized in the EPS matrix surrounding PAO cells in anaerobic and aerobic conditions respectively, as opposed to intra-cellularly [131]. Finally, a model of the physical structure of P-removing AGS has been proposed, where poly-phosphate was confirmed to be the dominant form of P within microbial cells as well as in the EPS matrix [155].

#### Cluster I: Characterization of activated sludge flocs

The mechanisms and factors that influence floc formation in suspended growth systems of heterotrophic bacteria with algae have been systematically reviewed [156]. Although the aim was to optimize the nutritional value and morphological characteristics for use as feed for aquaculture systems, such knowledge is applicable in conventional wastewater treatment, *e.g*. for improving sludge settle-ability as well as subsequent resource recovery applications. Sludge settle-ability in IFAS processes has been reported to be lower compared to suspended growth processes [157]. Interestingly, this was correlated to lower poly-phosphate content of the suspended relative to the attached phase, suggesting that the addition of IFAS media had a detrimental effect on EBPR. The last publication in this cluster reviewed the deployment of quantitative image analysis techniques for biomass characterization, with particular focus on identifying intra-cellular polymers involved in EBPR, notably poly-P, PHA and glycogen, and later on the microorganisms themselves via hybridization with fluorescent probes [158].

#### Cluster J: Biological phosphorus removal with pure cultures of *Acinetobacter*

Although the role of *Acinetobacter* in full-scale EBPR processes has been a subject of considerable debate, some research has focused on the attachment of pure cultures of *A. calcoaceticus* [159] and *A. junii* [160] on natural zeolite as a biofilm to improve P-removal.

#### Cluster K: Biological nutrient removal from aquaculture wastes

The deterioration of water quality due to excessive nutrient loading is of great concern in recirculating aquaculture systems, where P introduced in the feed is discharged with the organic solids and aqueous effluent. The oldest publication [161] presented evidence of denitrifying bacteria capable of storing poly-P, although not PHA, where simultaneous N and P-removal occurred under completely anoxic conditions. P-content was as much as 11.8% of the dry weight, which is inline with values reported for typical PAO. Although a number of different configurations have been compared [162], the expression of poly-phosphate related genes via meta-transcriptomics showed that it was sulfur-oxidizing bacteria rather than PAO that stored and hydrolyzed poly-P upon exposure to anoxic or sulfidic conditions, resulting in phosphate mineral formation [163]. As such, P-removal from recirculating aquaculture systems seems to be due to biologically-induced precipitation rather than EBPR.

#### Cluster L: Design, manufacture and application of micro-electrode sensors

Probing at different depths of a biofilm, micro-scale sensors revealed a delay between the beginning of the aerobic phase and the start of nitrification hypothesized as being due to competition between PAO and nitrifying bacteria for oxygen [164]. As such, in addition to the availability of carbon substrate, successful nitrification with EBPR was found to depend on a sufficiently long aerobic period to establish a nitrifying population.

### 3.5 Perspectives on applying the framework to conduct a literature review of EBPR

In this section we produced a bibliometric analysis and a review of the EBPR research domain, based solely on publications selected using the indicators developed in section 2. The initial clustering coupled with the keyword analysis allowed us to gain an immediate insight into the different research topics within EBPR. Analysis of citation profiles and dates of publication provided information on the temporal distribution of each topic and on whether they were declining or emerging. The number of highly-cited publications also indicated their overall impact. In each cluster, selecting only the most highly-cited publication each year and briefly analyzing their title, abstract and conclusions was sufficient to tell a fairly accurate story of the key developments of EBPR in its 40 years of history. Although the final content evaluation needed to be done manually, exercising a certain degree of critical sense, the number of selected publications constituted less than 5% of the original corpus. The same result would not have been possible by selecting the most cited publications in EBPR, due to the inevitable bias towards specific topics that have garnered more interest, *e.g*. cluster C, as well as by older publications that have had more time to accumulate citations, *e.g*. cluster A. Finally, the flow of information between clusters—how the topics evolved chronologically and inter-topically—could be inferred from the distribution of citations between different clusters.

One complication in the automated selection of key publications for review was the inclusion of papers seemingly unrelated to EBPR research, featuring *e.g*. phosphate adsorption [165, cluster G] or a review of mathematical models of the human gut ecosystem [166, cluster E]. Such publications did not feature any of the search terms applied in section 2.1, in either the title, abstract or keyword fields. This distortion was found to be due to the *KeyWord Plus* feature [167] which artificially tagged papers with keywords not listed by the original authors. This was intended to uncover publications that otherwise may not have been found due to changes in the use of scientific terms. Nevertheless, this feature does have merits, as exemplified by the contributions to metagenomic approaches applied to AS in cluster C [96, 168]. As there is no simple means to exclude results retrieved by *KeyWord Plus* terms, care must be exercised in the initial corpus assembly.

It must also be acknowledged that while clusters obtained via BC exhibited the highest degree of topical cohesion, the separation was not perfect. For instance, an article investigating the effects of titanium dioxide nano-particles on community shift in AS [169] was allocated to the topic of VFA generation from WAS (cluster F) instead of cluster G which dealt with community composition. It is possible that this was due to the consideration of enzymatic activity in addition to microbial community, as is common in publications related to fermentation processes for VFA production. Likewise, an investigation of the inhibitory effect of free nitrous acid (FNA) [170] was allocated to cluster D rather than with the other publications dealing with inhibition of P-uptake by nitrification-denitrification intermediates in cluster F. It could be argued that this was due to FNA inhibition of PAO metabolism being an important factor in their competition with GAO. The seemingly erroneous allocation may thus be attributed to topical overlap between two clusters.

Despite these challenges, the overall review is able to accurately describe, to our knowledge, the history of EBPR throughout its lifetime. In addition, while this framework was applied with the aim of presenting a review of key events, it can be adapted to suit other purposes. Each cluster can be subjected to further clustering to investigate sub-topics in greater depth. For instance, in our case cluster D could have been further subdivided to reduce the overlap between publications related to PHA production in mixed cultures and those that consider only the competition between PAO and GAO in EBPR systems. This would be particularly relevant in research areas with much larger volumes of publications. Further, the threshold definition may be varied in order to expand or contract the selection of highly-cited publications designated for review in each cluster. For instance, one may decide to review only the top cited publication in 5 year moving intervals, or alternatively select 5 publications in each year, allowing for a much higher level of resolution.

## Conclusions

This work proposed a framework for facilitating the selection of articles when conducting a literature review based on the combination of bibliometric techniques and other indicators applied to EBPR. In section 2, two clustering techniques were compared, bibliographic coupling and co-citation, with the former yielding the best results. The framework also included the use of keyword analysis, citation profiles, statistical analysis of dates of publication and benchmarks of citation counts as indicators to measure impact in the form of popularity, temporal trends and patterns in the flow of information. The fact that all this information can be statistically retrieved as opposed to relying uniquely on expert judgment, is the main advantage of using this framework for newcomers to a certain research field or even for experienced researchers to obtain a more systematic perspective. As a result of the framework, in section 3, twelve clusters were obtained, each equivalent to a topic under the umbrella of EBPR and a literature review was produced based on less than 5% of the size of the original EBPR corpus, meaning a less time-consuming approach.

The research domain of EBPR evolved in two principal ‘waves’: (1) correlation of operational conditions with process performance, theoretical frameworks for mechanisms and the identity and competition between the responsible organisms; (2) EBPR process intensification, optimization and further justification for the technology. Some topics are in decline, *e.g*. early developments of theoretical frameworks, metabolism of organisms relevant to EBPR, EBPR combined with MBR, and a number of smaller clusters on the characterization of AS flocs and the attachment of *Acinetobacter* on natural zeolite. Other topics are emerging, *e.g*. VFA production from WAS for simultaneous N and P-removal, P-recovery for the circular economy, and aerobic granular sludge for better treatment efficiency and process stability. Although BNR from aquaculture wastes is also an emergent area of research, P-removal was a result of biologically-induced precipitation rather than EBPR. Topics which have had the highest impact include the genetic profiling of microbial communities in EBPR systems, the competition between PAO and GAO, VFA generation and P-recovery from WAS, as well as EBPR using aerobic granules.

The development and application of this framework to conduct a literature review of EBPR achieved two main outcomes. First, we provided insights into the evolution of a multi-disciplinary area of research, detected its emerging areas and outlined the key events throughout its development. Second, with increasing rates of scientific publication and information dissemination, we hope to have contributed a step in the direction of systematic and automated curation of knowledge repositories.

## Supporting information

S1 File Node list. Publications with corresponding identifiers, including cluster membership. Generated as a ‘map file’ using *VOSviewer*.
(TXT)

S2 File Adjacency list. Description of non-directional edges between pairs of publications, including edge strength information. Generated as a ‘network file’ using *VOSviewer*.
(TXT)

S3 File Adjacency matrix. Square representation of edges between different clusters in the bibliographic coupling network, corresponding to Fig 7.
(TXT)

S4 File Data processing template for Python 3. (PY)

S1 Fig Citation profile distributions. Distribution of peaks from the citation profile analysis, excluding (left plot) and including (right plot) multi-peaks. The data, shown as histograms, was modelled using the Gaussian distribution (red curve) to estimate the mean (*μ*) and standard deviation (*σ*). The boxplots illustrate the mean (red), median (dashed-green) and interquartile range (whiskers).
(EPS)
