# Correction: New framework for automated article selection applied to a literature review of Enhanced Biological Phosphorus Removal

**DOI:** 10.1371/journal.pone.0218142

**Published:** 2019-06-06

**Authors:** Minh Nguyen Quang, Tim Rogers, Jan Hofman, Ana B. Lanham

The first author’s initials appear incorrectly in the citation. The correct citation is: Nguyen Quang M, Rogers T, Hofman J, Lanham AB (2019) New framework for automated article selection applied to a literature review of Enhanced Biological Phosphorus Removal. PLoS ONE 14(5): e0216126. https://doi.org/10.1371/journal.pone.0216126
